# Post-traumatic stress disorder symptoms and associated factors in breast cancer patients during the first COVID-19 lockdown in France

**DOI:** 10.3389/fpsyg.2022.768043

**Published:** 2022-09-15

**Authors:** Feriel Yahi, Justine Lequesne, Olivier Rigal, Adeline Morel, Marianne Leheurteur, Jean-Michel Grellard, Alexandra Leconte, Bénédicte Clarisse, Florence Joly, Sophie Lefèvre-Arbogast

**Affiliations:** ^1^Clinical Research Department, Centre François Baclesse, Caen, France; ^2^Department of Health, University of Caen Normandie, Caen, France; ^3^Department of Medical Oncology, Centre Henri Becquerel, Rouen, France; ^4^Department of Medical Oncology, Centre François Baclesse, Caen, France; ^5^INSERM U1086 ANTICIPE, Caen, France

**Keywords:** psychological distress, breast cancer, COVID-19, post-traumatic stress disorder (PTSD), quality of life

## Abstract

**Introduction:**

We aimed to study post-traumatic stress disorder (PTSD) symptoms in breast cancer (BC) patients during the coronavirus disease (COVID-19) pandemic.

**Materials and methods:**

We included BC patients receiving medical treatment during the first COVID-19 lockdown in France. PTSD symptoms were evaluated using the Impact of Event Scale-Revised (IES-R) questionnaire. Quality of life [Functional Assessment of Cancer Therapy-General (FACT-G)], cognitive complaints [Functional Assessment of Cancer Therapy–Cognitive Function (FACT-Cog)], insomnia [Insomnia Severity Index (ISI)], and psychosocial experiences during lockdown were also evaluated. Multivariable logistic regression was used to identify clinical factors (from medical records) and psychosocial factors (from questionnaires) associated with PTSD symptoms.

**Results:**

Among the 253 included BC patients (mean age: 58), 46% had metastatic cancer and 52% were treated by chemotherapy alone. COVID-19-induced adjustments in medical oncology practices were experienced by 27% of patients (mainly teleconsultations). No case of COVID-19 was reported; 23% of BC patients had PTSD symptoms. Compared to other patients, patients with PTSD symptoms had more fears relative to COVID-19 infection (83 vs. 60%, *p* = 0.009), had more feeling of isolation (69 vs. 41%, *p* = 0.003), and had more prescription or increased use of psychotropic drugs (49 vs. 20%, *p* = 0.001). In the multivariable model adjusted for clinical factors, fears relative to COVID-19 and increased use of psychotropic drugs were independently associated with PTSD symptoms (OR [95% CI] = 3.01 [1.20–8.44] and 3.45 [1.48–8.17], respectively). Besides, patients with PTSD symptoms had poor quality of life (QoL), and more cognitive complaints and insomnia.

**Conclusion:**

Post-traumatic stress disorder symptoms were observed in 23% of BC patients during the first COVID-19 lockdown in France. Psychological supports are needed for patients treated during the COVID-19 pandemic.

## Introduction

Since December 2019, the coronavirus disease (COVID-19) has a major impact on the healthcare system and social life around the world. On 17 March 2020, a nationwide lockdown was implemented in France in order to prevent the disease from spreading (Cauchemez et al., 2020).

The COVID-19 pandemic and associated social restrictions have deteriorated the population’s psychological health, causing fears of infection, anxiety, and stress (French et al., 2020; Salari et al., 2020; Chen et al., 2021). It has been described as a traumatic stressor that may trigger symptoms of post-traumatic stress disorder (PTSD), especially in women and people at high risk of severe infection (Bridgland et al., 2021). Breast cancer (BC) patients might be particularly vulnerable to such psychological distress (Bargon et al., 2021). Owing to their weakened immune system and their poor survival outcomes when infected by the COVID-19 (Liang et al., 2020), cancer patients, especially female patients, have developed fears to be infected by the COVID-19 (Sigorski et al., 2020; Sutcuoglu et al., 2020). In addition, they have been deprived of social support from family and friends which led to social isolation and loneliness (Gallagher et al., 2020; Biagioli et al., 2021). Moreover, the pandemic induced unexpected and challenging problems to the routine delivery of BC treatment management and supportive healthcare (Chan et al., 2020; Soran et al., 2020). Numerous guidelines have been developed to adjust BC medical protocols (Curigliano et al., 2020; de Azambuja et al., 2020; Manoj Gowda et al., 2020). Surgical treatments were delayed when possible, different forms of treatment were modified, and monitoring visits were canceled, postponed, or turned into video calls. This led to fears of cancer care disruption (Guven et al., 2020).

The psychological health of BC patients has been described as very deteriorated in literature, as reported by a meta-analysis of Hashemi et al. (2020). Especially, PTSD symptoms are frequently observed at diagnosis and may persist over time for half of the patients (O’Connor et al., 2011; Voigt et al., 2017). Few studies have investigated the psychological health of BC during the COVID-19 pandemic, which may be considered as a potential additional stress factor. A study from the Netherlands observed deterioration in emotional functioning and moderate to severe loneliness in patients (Bargon et al., 2021). A British study suggested that the pandemic-induced disruption to scheduled oncology services had a negative impact on cognitive function (Swainston et al., 2020). A French study measured PTSD and anxiety in cancer patients in France following the first COVID-19-related lockdown and associated factors (Marino et al., 2022).

To our knowledge, only two studies explored PTSD symptoms in BC patients at the very beginning of the COVID-19 pandemic in China and found that BC experienced high rates of anxiety, depression, distress, and insomnia (Cui et al., 2020; Juanjuan et al., 2020). This study aimed to estimate the rate of PTSD symptoms among BC patients during the first COVID-19 lockdown in France and to identify the associated clinical and psychosocial factors.

## Materials and methods

### Study design and participants

COVIPACT is a prospective French study that enrolled 734 participants who were at least 18 years old, were treated for solid or hematologic cancer in two regional cancer centers (Centre François Baclesse, Caen and Centre Henri Becquerel, Rouen) during the first COVID-19 lockdown, and had given their written consent to participate. The study’s primary objective was to describe the pandemic-induced adjustments in medical oncology practice, as well as the psychological distress and quality of life (QoL) of cancer patients during this period. Previously published results showed that 21% of cancer patients experienced PTSD associated with poor QoL during the first lockdown (Joly et al., 2021). In this study, we aimed to investigate those outcomes in the sub-population of BC patients, which represents 304 patients of the sample. Patients who were at least 18 years old and who were receiving an oncological treatment for BC between 17 March 2020 (beginning of the first nationwide lockdown) and 29 May 2020 at the daycare hospital were included. Patients exclusively receiving oral therapy of simple surveillance were excluded.

The research received ethical approval from the local ethics committee (ID RCB: 2020-A00879-30). The data that support the findings of this study are available from the corresponding author upon reasonable request.

### Data collection

Demographics and clinical data, including age, body mass index, Eastern Cooperative Oncology Group (ECOG) performance status, and stage of the disease, as well as adjustments in medical oncology practice during the lockdown, were collected from medical records (April to May 2020). Simultaneously, patients completed validated questionnaires to assess their PTSD symptoms [Impact of Event Scale-Revised (IES-R)], QoL [Functional Assessment of Cancer Therapy-General (FACT-G)], cognitive complaints [Functional Assessment of Cancer Therapy–Cognitive Function (FACT-Cog)], and sleep quality [Insomnia Severity Index (ISI)]. This time point is defined as baseline, which corresponds to around 1 month after the beginning of COVID-19 lockdown. Each questionnaire’s responses were rated on a 5-point Likert scale from 0 (not at all) to 4 (extremely). Three months after baseline (Summer 2020), patients completed a questionnaire to retrospectively evaluate their social and psychological experiences during the lockdown.

#### Impact of event scale-revised

The IES-R is a self-administered questionnaire that assesses a person’s subjective response to a traumatic event and may indicate PTSD symptoms (Weiss, 2007). As the authors of the IES-R allow instructions for the assessed event, in our study, patients were asked to complete the questionnaire considering the first COVID-19 lockdown as the traumatic event, in its French-validated form (Brunet et al., 2003). The IES-R is made up of 22 items that were grouped into three subscales, namely, avoidance, intrusion, and hyperarousal. The total score ranges from 0 to 88, and a total IES-R score ≥33 indicates PTSD symptomatology (Creamer et al., 2003).

#### The functional assessment of cancer therapy–general

The FACT-G consists of 27 items grouped into four QoL domains, namely, physical (PWB), social/family (SWB), emotional (EWB), and functional (FWB) wellbeing (Cella et al., 1993; Conroy et al., 2004). The PWB subscale assesses physical concerns, the SWB subscale assesses social communications, the EWB subscale assesses mood as well as mental responses to illness, and the FWB subscale assesses a person’s ability to perform a variety of activities. The total score ranges from 0 to 108, with higher scores indicating higher QoL.

#### The functional assessment of cancer therapy–cognitive function

The Functional Assessment of Cancer Therapy-Cognitive Scale is a 37-item self-report inventory that assesses all perceived cognitive impairments and cognitive abilities over the previous 7 days (Joly et al., 2012). The total FACT-Cog score is divided into four subscales, namely, Perceived Cognitive Impairments (20 items; score range 0–72), Impact on QoL (4 items; score range 0–16), Comments from Others (4 items; score range 0–16), and Perceived Cognitive Abilities (9 items; score range 0–28).

#### Insomnia severity index

The insomnia severity index (ISI) is a 7-item self-report questionnaire that evaluates the nature, severity, symptoms, and consequences of insomnia (Blais et al., 1997; Morin et al., 2011). The total score ranges from 0 to 28 and can be categorized into absence of insomnia (0–7), sub-threshold insomnia (8–14), moderate insomnia (15–21), and severe insomnia (22–28).

#### The COVID-19 social and psychological experiences questionnaire

This questionnaire assessed the social and psychological experiences during the first COVID-19 lockdown. In the first section on the social structure of the lockdown, patients were asked whether the patient lived in an apartment or a house, worked or not, spent the lockdown alone or with others (spouses, children, relatives, or parents), and had any physical or virtual social relationships. In the second section regarding psychological experiences during the lockdown, patients scored their feeling of isolation and fears of COVID-19 infection (four items: fear to be infected during the hospital treatment, fear to be infected during outing to purchase necessities, fear to infect a close person, and fear to have a close person infected) on a 4-point Likert scale that we dichotomized for analysis. In addition, patients were asked if any of the following psychotropic treatments were prescribed or increased during the lockdown: anxiolytics, antidepressants, analgesics, and sleeping pills or hypnotics.

### Statistical analysis

The rate of BC patients with PTSD symptoms during the first COVID-19 lockdown was estimated with a 95% confidence interval (CI). The characteristics of patients were described as numbers (%) for qualitative variables and mean (±standard deviation) [min–max] unless specified otherwise for quantitative variables. We used Student’s *t*-test, chi-square test, and Wilcoxon test to compare clinical and psychological factors between patients with and without PTSD symptoms. To determine the factors independently associated with PTSD symptoms, we carried out a multivariable logistic regression model. We included preselected clinical factors (age, stage, and time since diagnosis), study center, and all other factors associated with PTSD symptoms in univariate comparisons. In secondary analyses, scores of QoL, insomnia, and cognitive complaints were described and compared by PTSD symptoms using Student’s *t*-test or chi-square test. A *p*-value of <0.05 was considered statistically significant. R software (version 4.0.3) was used for all statistical analyses.

## Results

Among the 304 patients with BC from the COVIPACT study, we included 253 patients who completed questionnaires on PTSD symptoms, QoL, insomnia, and cognition. Among them, 183 (72%) patients also completed the COVID-19 social and psychological experiences questionnaire.

### Demographic and clinical characteristics of patients

Of the 253 BC patients included in our analysis, 82% of patients were aged under 70 years, the majority had an ECOG score under two (98%), and 46% of patients had metastatic cancer (Table 1). Most patients had initiated their treatment before lockdown (65%), and chemotherapy alone was the most common treatment administered to patients (52%).

**TABLE 1:** Clinical characteristics of breast cancer (BC) patients (*N* = 253).

Clinical characteristics of patients	*N* (%) or mean (SD) [min–max]
Age (years-old), mean (SD) [min–max]	58 (12) [29–89]
Age < 70	207 (82)
Age ≥ 70	46 (18)
BMI (kg/m^2^), mean (SD) [min–max]	26 (5) [17–44]
ECOG	
0	68 (27)
1	179 (71)
≥2	4 (2)
Stage	
Metastatic	116 (46)
Localized	137 (54)
Time since diagnosis (months), mean (SD) [min–max]	51 (76) [1–371]
*De novo* treatment*	
Yes	149 (59)
No	104 (41)
Initiation of treatment	
Before lockdown	160 (65)
After lockdown	85 (35)
Current therapy	
Chemotherapy alone	131 (52)
Targeted therapy alone	71 (28)
Chemotherapy and targeted therapy/immunotherapy	30 (12)
Other treatment	20 (8)
History of chronic conditions	
Hypertension	67 (26)
Cardiovascular disease	33 (13)
Psychological disorders	26 (10)
Pulmonary disease	22 (9)
Other cancer	19 (7)
Diabetes	17 (7)
Immune disease	4 (2)
Kidney disease	3 (1)
Other	60 (24)

Values are *N* (%) of non-missing data, unless specified otherwise. Data are missing for <4% of patients (8 missing Initiation of treatment, 2 missing ECOG, and 1 missing current therapy). *The first line of treatment for breast cancer (BC) as opposed to recurrence treatment or refractory.

### Adjustment in medical oncology practices

During the first COVID-19 lockdown, adjustments in medical oncology practices were experienced by 27% of patients, with adapted monitoring (teleconsultation) being the most frequent adjustment (59%) (Supplementary Table 1). Adjustments mainly concerned patients treated by targeted therapies (42%) who experienced teleconsultations, interruption, and adjournment of their treatments (37, 27, and 57%, respectively).

### The COVID-19 social and psychological experiences questionnaire

Among the 183 patients who responded to the questionnaire related to the COVID-19 first lockdown, 16% of them spent this period alone and 25% had no social interaction (Table 2). During this lockdown, 65% of the patients had fears about COVID-19 infection and 48% felt socially isolated. Few patients were receiving supportive care before the lockdown (24%, 44/183), and most of them were discontinued during the lockdown (66%, 29/44). Besides, 27% of patients indicated having prescription or increased use of psychotropic drugs.

**TABLE 2:** The social and psychological experiences of breast cancer (BC) patients during the first coronavirus disease (COVID-19) lockdown (*N* = 183).

	*N* (%)
Residence during lockdown	
Apartment	24 (13)
House	158 (87)
Occupational job status	
Active	66 (37)
Retired	71 (39)
No activity	44 (24)
Distance between home and health facility (km) median [min–max]	40 [0–190]
Living conditions	
Alone	28 (16)
With adults only (spouse/relatives)	84 (48)
With children and/or adults	64 (36)
Social interactions	
Virtual	112 (62)
Physical	24 (13)
None	44 (25)
Contact with infected person	27 (15)
Fears relative to COVID-19 infection	119 (65)
Feeling of isolation	87 (48)
Prescription or increased use of psychotropic drugs	46 (27)

Values are *N* (%) of non-missing data, unless specified otherwise. Data are missing for <4% of patients (2 missing occupational job status, 7 missing living conditions, 3 missing social interactions, 5 missing contact with infected person, 1 missing fear relative to COVID-19 infection, and 10 missing prescription or increased use of psychotropic drugs).

### Post-traumatic stress disorder symptoms and associated factors

**FIGURE 1:**
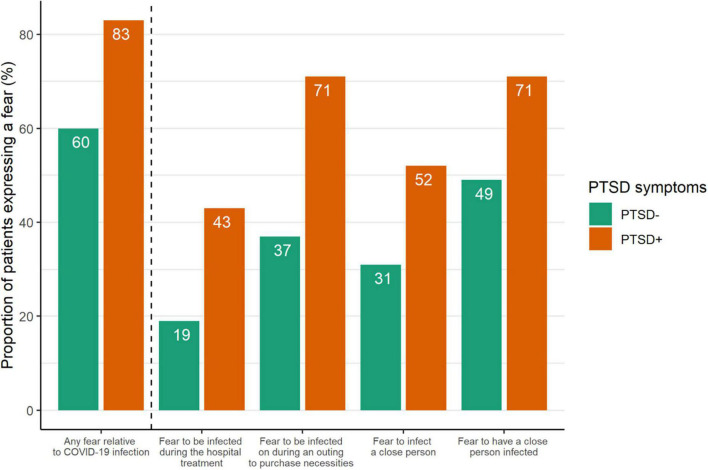
Proportion of breast cancer (BC) patients expressing fears relative to coronavirus disease (COVID-19) infection by post-traumatic stress disorder (PTSD) symptoms (*N* = 183). Date are issued from the questionnaire which assessed social and psychological experiences during the first COVID-19 lockdown. Patients scored their fears of COVID-19 infection (4 items: fear to be infected during the hospital treatment, fear to be infected during outing to purchase necessities, fear to infect a close person, and fear to have a close person infected) on a 4-point Likert scale that were dichotomized for analysis (“not at all/yes, a little” vs. “yes, moderately/yes, a lot”).

Post-traumatic stress disorder symptoms were reported by 58 patients (23%: 95% CI [18–28.7]). The mean IES-R score was 22 in our population (mean intrusion, avoidance, and hyperarousal subscales = 9, 9, and 5, respectively; results not in tables). No clinical factor was significantly associated with PTSD symptoms in our sample (Table 3). However, patients with PTSD symptoms reported significantly more fears relative to COVID-19 than patients without PTSD symptoms (83 vs. 60%, *p* = 0.009; Figure 1). Fear to be infected during an outing to purchase necessities and fear to have a close person infected were highly expressed by patients with PTSD symptoms (71% for both). Furthermore, as compared to other patients, patients with PTSD symptoms felt more socially isolated (69 vs. 41%, *p* = 0.003; Supplementary Table 2) and had more prescription or increased use of psychotropic drugs (49 vs. 20%, *p* = 0.001; Supplementary Table 2).

**TABLE 3:** Characteristics of patients by post-traumatic stress disorder (PTSD) symptoms (*N* = 253).

	PTSD symptoms (IES-R ≥33) *N* = 58	No PTSD symptoms (IES-R <33) *N* = 195	*P*-value
Age (years-old), mean (SD)	58 (13)	58 (12)	0.83
Age ≥ 70	50 (86)	157 (80)	0.42
Age < 70	8 (14)	38 (20)	
BMI (kg/m^2^) mean (SD)	26 (5)	26 (5)	0.73
ECOG			0.91
0	14 (25)	54 (28)	
≥1	42 (75)	141 (72)	
Stage			0.35
Metastatic	23 (40)	93 (48)	
Localized	35 (60)	102 (52)	
Time since diagnosis (months), mean (SD)	47 (74)	53 (76)	0.64
*De novo* treatment			0.48
Yes	37 (64)	112 (57)	
No	21 (36)	83 (43)	
Initiation of treatment			0.93
Before lockdown	19 (33)	66 (35)	
After lockdown	38 (67)	122 (65)	
Current therapy			0.36
Chemotherapy alone	34 (59)	97 (50)	
Targeted therapy alone	14 (24)	57 (29)	
Chemotherapy and targeted therapy/immunotherapy	4 (7)	26 (13)	
Other treatment	6 (10)	14 (7)	
Psychological disorders	6 (10)	20 (10)	1
Any treatment adjustment during lockdown	18 (31)	50 (26)	0.52

Values are *N* (%) of non-missing data, unless specified otherwise. *p*-values are from Student’s *t*-test or chi-square test. Data are missing for <4% of patients (8 missing initiation of treatment, 2 missing ECOG, and 1 missing current therapy).

In the multivariable model adjusted for age, cancer stage, time since diagnostic, and study center, patients who had fears relative to COVID-19 and had a prescription or an increased use of psychotropic drugs were 3 times more likely to have PTSD symptoms than other patients (OR [95% CI] = 3.01 [1.20–8.44]; OR [95% CI] = 3.45 [1.48–8.17], respectively; Table 4).

**TABLE 4:** Multivariable model of post-traumatic stress disorder (PTSD) symptoms during the first coronavirus disease (COVID-19) lockdown in breast cancer (BC) patients (*N* = 183).

	OR [95% CI]	*P*-value
Age		0.75
<70	1.18 [0.39–3.36]	
≥70	1	
Stage		0.87
Localized	1	
Metastatic	1.07 [0.40–2.82]	
Time since diagnosis, for 10 month-increase	0.96 [0.90–1.02]	0.27
Fears relative to COVID-19		**0.02**
No	**1**	
Yes	**3.01 [1.20–8.44]**	
Feeling of isolation during lockdown		0.12
No	1	
Yes	1.94 [0.83–4.50]	
Prescription or increase of psychotropic drugs		**0.004**
No	**1**	
Yes	**3.45 [1.48–8.17]**	

Model is adjusted for study center. OR, Odds ratio 95%; CI, confidence interval. Bold values refer to the significant results at 5% alpha level.

### Insomnia, quality of life, cognition, and post-traumatic stress disorder symptoms

Among the 253 BC patients, 28% had moderate to severe insomnia (Table 5), which was more often observed in patients with PTSD symptoms than in other patients (55 vs. 20%, *p* < 0.001).

**TABLE 5:** Insomnia, quality of life (QoL), and cognitive complaints by post-traumatic stress disorder (PTSD) symptoms (*N* = 253).

	Total *N* = 253	PTSD symptoms (IES-R ≥ 33) *N* = 58	No PTSD symptoms (IES-R < 33) *N* = 195	*P*-value
**ISI**				
Total score	10 (7)	16 (5)	9 (6)	**<0.001**
ISI categories *N* (%)				**<0.001**
No insomnia (0–7)	97 (38)	4 (7)	93 (48)	
Little insomnia (8–14)	85 (34)	22 (38)	63 (32)	
Moderate to severe insomnia (15–28)	71 (28)	32 (55)	39 (20)	
**FACT-G**				
Total score	76 (14)	64 (13)	80 (12)	**<0.001**
Physical subscale (PWB)	21 (5)	17 (6)	22 (4)	**<0.001**
Social subscale (SWB)	22 (5)	21 (5)	21 (5)	0.80
Emotional subscale (EWB)	17 (5)	13 (5)	19 (4)	**<0.001**
Functional subscale (FWB)	16 (5)	13 (5)	17 (5)	**<0.001**
**FACT-Cog**				
Perceived Cognitive Impairment (PCI)	60 (12)	51 (15)	63 (10)	**<0.001**
Perceived Cognitive Abilities (PCA)	20 (6)	10 (3)	12 (3)	**0.001**
Impact on Quality of Life (QoL)	11 (5)	8 (4)	12 (4)	**<0.001**
Comments from Others (Oth)	15 (2)	15 (3)	16 (1)	**<0.001**

Values are mean (SD), unless specified otherwise; *p*-values are from Student’s *t*-tests or chi-square tests. Bold values refer to the significant results at 5% alpha level.

The mean total score of the FACT-G was 76 (±14). Patients with PTSD symptoms had a lower FACT-G score (indicating worse QoL) than patients without PTSD symptoms [mean 64 (±13) vs. 80 (±12), *P* = 0.001]. All FACT-G subscales were substantially lower in patients with PTSD symptoms compared to patients without PTSD symptoms, except the social subscale. Similarly, patients with PTSD symptoms had significantly lower scores for all dimensions of cognitive function as assessed by the FACT-Cog.

## Discussion

In our study, we observed that 23% of BC patients had PTSD symptoms during the first lockdown in France. Fears relative to COVID-19 infection, feeling of isolation, and prescription or increased use of psychotropic drugs during lockdown (reported by 65, 48, and 27% of the patients, respectively) were associated with more frequent PTSD symptoms. Besides, we observed poor QoL, cognitive function, and sleep quality in patients with PTSD symptoms compared to patients without PTSD symptoms.

Several studies reported poor mental health and high level of anxiety and distress in the population during the COVID-19 pandemic. Two studies showed that 16.3 and 23.6% of the general population had PTSD symptoms during the COVID-19 pandemic (as measured with the IES-R Questionnaire) (Alkhamees et al., 2020; Tee et al., 2020). Generally, a systematic review reported that COVID-19 was associated with psychological distress, especially for women, with however a great heterogeneity in PTSD symptoms rates (from 7 to 53.8%) (Xiong et al., 2020). Among cancer patients, it was reported 36% of PTSD symptoms in lymphoma patients during the onset of COVID-19 in Italy (Romito et al., 2020) and 31% in an American study of mostly BC patients (Miaskowski et al., 2020). A French oncological survey identified moderate-to-severe PTSD in 14.7% of cancer patients during the first pandemic COVID-19 lockdown, with a higher rate of 18% in the breast or gynecological patients (Marino et al., 2022). Two Chinese studies that assessed the prevalence of PTSD symptoms very early in the course of the COVID-19 pandemic found 35.5 and 52.2% moderate to severe distress symptoms in BC patients (Cui et al., 2020; Juanjuan et al., 2020). Overall, our findings were comparable to those found in cancer patients’ studies that generally found a higher frequency of PTSD symptoms than that in the general population.

According to our study, half of BC patients felt isolated during the lockdown, two-third had fears relative to the COVID-19 infection, and both factors were associated with PTSD symptoms. In agreement, a qualitative study described that the lockdown was very stressful for BC patients because they had to limit their outings and interactions with family and friends, and because they were concerned about catching the virus, especially when going to the hospital (Savard et al., 2021). Social isolation, as measured by a dedicated scale on emotional and social loneliness, was also observed in 48% of BC patients in a prospective Dutch study (Bargon et al., 2021). In this study, they also reported a significant drop in the social functioning of actively treated patients during the COVID-19 when compared to a pre-COVID-19 period (Bargon et al., 2021). Regarding fears of COVID-19 infection, varying levels of worry were found in previous studies in cancer patients, ranging from 19.8 to 65.6% (Guven et al., 2020; Juanjuan et al., 2020). Especially, it has been shown in general European populations and cancer population that women had a significantly higher level of fear of COVID-19 than men (Reznik et al., 2020; Sigorski et al., 2020). As a result, among cancer patients, the highest level of fear and anxiety was observed in patients with BC (Sigorski et al., 2020). However, it was suggested that actively treated patients with cancer were more concerned about cancer than about COVID-19 infection (Sigorski et al., 2020; Patni et al., 2021), both fears being closely linked. Indeed, some studies reported that BC patients were concerned about the efficacy of delayed/adapted therapies (Juanjuan et al., 2020). In other studies, BC patients interrupted their treatment because of fears of contracting COVID-19 in hospitals, resulting in changes and delays in their treatments (Alipour et al., 2020; Papautsky and Hamlish, 2020; Villarreal-Garza et al., 2021).

A third of our patients had a prescription or an increased use of psychotropic drugs. Several studies, including some in the general population, evaluated the use of psychotropic drugs during the period of the pandemic. Two studies conducted at the pandemic onset (between February and March 2020) in the United Kingdom and the United States found a considerable increase in the use of psychotropic drug prescriptions compared to 2019 (Stettin, 2020; Rabeea et al., 2021). Moreover, when the lockdown was declared in the United Kingdom, medication interventions increased by two per week, with hypnotics and benzodiazepines being the most commonly prescribed psychotropic medications (Rauf et al., 2021). Moreover, García-Fernández et al. (2020) found that the use of anxiolytics was associated with higher emotional distress in the elderly, which is in line with our results.

In the entire COVIPACT cohort, cancer patients who experienced an adjustment in medical oncology practices were found to be more vulnerable to PTSD symptoms (Joly et al., 2021). In this study focused on the sub-population of BC patients, we found that 27% of patients experienced adjustment in medical oncology practices during the lockdown, but it was not associated with more PTSD symptoms. This discrepancy in our global population might relate to the type of adjustment that was implemented. In BC patients, the most common adjustment was the implementation of teleconsultation, which may be less troubling for patients than rescheduled or rearranged treatments. Indeed, two studies aiming to assess the relationship between delaying treatment and deterioration of cancer patients’ mental health observed that treatment delay or interruption was among the dominant factors contributing to anxiety and depression (Chen et al., 2020; Ciazynska et al., 2020).

Finally, our results indicated that patients with PTSD symptoms presented worse QoL, more cognitive complaints, and more insomnia compared to patients without PTSD symptoms. Similarly, Miaskowski et al. (2020) found that cancer patients with PTSD symptoms reported clinically meaningful levels of depressive symptoms, anxiety, pain, cognitive complaints, and sleep disturbance.

There were some limitations in this study. First, this is a cross-sectional analysis of the COVIPACT study in which no data were collected before lockdown, which precluded to properly assess that PTSD symptoms emerged from the COVID-19 (and associated social restrictions) stressor. O’Connor et al. (2011) showed that receiving a BC diagnosis could also be a significant traumatic experience and that many women experienced persistent cancer-related PTSD symptoms. This suggests that PTSD symptoms could be associated with cancer rather than with COVID-19. However, in our study, only factors related to COVID-19 (e.g., fears of infection and social isolation), but no cancer-related factors (e.g., stage of cancer; time since diagnosis), were associated with PTSD symptoms, which is in support of the contribution of the COVID-19 context to the deterioration of psychological health in cancer patients. Second, the IES-R Questionnaire is not a formal diagnostic tool, but it is widely used to detect symptoms of psychological distress. Finally, although most patients-reported outcomes were assessed using validated questionnaires, social isolation was assessed using a single question of a non-validated questionnaire, rather than a specific and validated scale (Sarason et al., 1987). Despite these limitations, our study is one of few studies that examined specifically PTSD symptoms in BC patients and explored their association with both clinical factors (collected from medical records) and psychosocial factors during the first COVID-19 lockdown. The recruitment period coincides specifically with the date of the first lockdown (from 17 March to 3 May 2020). In the beginning, only 7,730 cases of COVID-19 were observed in France but the spread of the virus and the high rate of death (175 among 7,730) may contribute to the severity of anxiety and PTSD. Moreover, the PTSD assessment has been performed around 1 month after the start of the lockdown, which is a sufficient time duration to observe the true effect of lockdown on PTSD symptoms.

Overall, according to our study, 23% of BC patients experienced PTSD symptoms during the first COVID-19 lockdown in France. While no clinical factors were associated with PTSD symptoms, patients who had fears relative to COVID-19, feeling of social isolation, and prescription or increased use of psychotropic drugs were more likely to express PTSD symptoms, supporting that the COVID-19 pandemic, beyond cancer, may contribute to psychological distress in this BC population. This study can contribute to suggest additional psychological support during this long period of pandemic. COVIPACT 2 (NCT04747249), a study including the cancer patients of COVIPACT study identified with a moderate to severe level of PTSD, is underway to assess the benefit of psychological interventions to reduce them.

## Data availability statement

The raw data supporting the conclusions of this article will be made available by the authors, without undue reservation.

## Ethics statement

The studies involving human participants were reviewed and approved by ref. 220 C07; South Mediterranean II Committee for the Protection of Persons. The patients/participants provided their written informed consent to participate in this study.

## Author contributions

FY: bibliography, statistical analysis, and writing the manuscript. JL: conceptualization, methodology, results interpretation, help for redaction, supervision, and submission. OR: resources and project administration. AM and ML: resources. J-MG and AL: project administration. BC: conceptualization, methodology, funding acquisition, and supervision. FJ: conceptualization, results interpretation, help for redaction, and supervision. SL-A: bibliography, results interpretation, help for redaction, and supervision. All authors contributed to the article and approved the submitted version.
