# Supplementary material for: Post-traumatic stress disorder symptoms and associated factors in breast cancer patients during the first COVID-19 lockdown in France
Source: Front Psychol. 2022 Sep 15;13:768043. doi: 10.3389/fpsyg.2022.768043 (PMC9521190; doi:10.3389/fpsyg.2022.768043)
Supplement: Supplementary file 1 [file Data_Sheet_1.docx]

**Supplementary Table 1: Details of adjustment in medical oncology practices during the first COVID-19 lockdown by current therapy**

|  | Any adjustment, N (% by therapy) | Type of adjustment, N | | | | |
| --- | --- | --- | --- | --- | --- | --- |
|  |  | **Adapted monitoring** | **Interruption** | **Adjournment** | **Type of treatment** | **Other** |
| Total; N=253 | 68 (27) | 40 | 9 | 19 | 3 | 3 |
| CT alone; N=131 | 21 (16) | 16 | 0 | 1 | 2 | 2 |
| TT alone; N=71 | 30 (42) | 11 | 8 | 17 | 0 | 0 |
| CT and TT/IT; N=30 | 7 (23) | 5 | 1 | 0 | 1 | 0 |
| Other; N=20 | 10 (50) | 8 | 0 | 1 | 0 | 1 |

Abbreviations: CT: Chemotherapy; TT: Target therapy; IT: Immunotherapy

**Supplementary Table 2: The social and psychological experiences of patients during the first COVID-19 lockdown by post-traumatic stress disorder (PTSD) symptoms (N=183)**

|  | PTSD symptoms  (IES-R≥ 33) N=42 | No PTSD symptoms  (IESR<33) N=141 | p-value |
| --- | --- | --- | --- |
| Residence during lockdown  Apartment  House | 5 (12)  37 (88) | 19 (14)  121 (86) | 0.98 |
| Occupational job status  Active  Retired  No activity | 14 (33)  17 (41)  11 (26) | 52 (37)  54 (39)  33 (24) | 0.88 |
| Distance between home and health facility (km), median | 46 | 40 | 0.58 |
| Living conditions  Alone  With adults only (spouse/relatives)  With children and/or adults | 7 (17)  20 (48)  15 (36) | 21 (16)  64 (48)  49 (37) | 0.99 |
| Social Interactions  Virtual  Physical  None | 25 (63)  6 (15)  9 (22) | 87 (62)  18 (13)  35 (25) | 0.91 |
| Contact with infected person | 6 (15) | 21 (15) | 1 |
| Fears relative to COVID-19 infection | **35 (83)** | **84 (60)** | **0.009** |
| Feeling of isolation | **29 (69)** | **58 (41)** | **0.003** |
| Prescription or increased use of psychotropic drugs | **20 (49)** | **26 (20)** | **0.001** |

Values are N (%) of non-missing data, unless specified otherwise. P-values are from Wilcoxon or Chi² tests. Data are missing for <4% of patients (2 missing occupational job status, 7 missing living conditions, 3 missing social interactions, 5 missing contact with infected person and 1 missing fear relative to COVID-19 infection, 10 missing prescription or increased use of psychotropic drugs).
